# Partial Duplication of the Left Ureter and Enlarged Left Suprarenal Gland – A Case Report

**DOI:** 10.7759/cureus.20848

**Published:** 2021-12-31

**Authors:** Bharat Sontakke, Vishwajit Deshmukh, Kirubhanand C, Gayatri Muthiyan, Gugapriya TS, Aaditya Tarnekar

**Affiliations:** 1 Anatomy • Genetics, All India Institute of Medical Sciences, Nagpur, IND; 2 Anatomy • Neuroanatomy, All India Institute of Medical Sciences, Nagpur, IND; 3 Anatomy, All India Institute of Medical Sciences, Nagpur, IND

**Keywords:** anomalies, left suprarenal gland, duplication, left kidney, left ureter

## Abstract

During routine dissection classes for the first-year undergraduate medical students, we encountered an anatomical variation concerning the duplication of the ureter. Usually, a single ureter connects to the posterior renal hilum. In the present case, a double ureter arises from the hilum on the left side and an enlarged suprarenal gland. The accessory ureter travels a short distance from the hilum before joining the other ureter. These anomalies are becoming more common during renal surgeries and renal transplant surgeries. Damage to the accessory ureter may result in complications following surgery. Knowledge of an accessory ureter and an enlarged suprarenal gland is clinically important for renal surgeons, radiologists, and anatomists.

## Introduction

With the increase of interventional radiological procedures, vascular operations, urological procedures, and renal transplantations, findings of renal tract variations are more common [1]. Each ureter is a cylindrical tube with a thick tunica media, and it measures 25-30 cm in length with an average 3-4 mm diameter. Each ureter is continuous with the funnel-shaped renal pelvis (5-7 ml capacity) located within the kidney. The ureter travels from the renal pelvis into the abdominal and pelvic cavities and enters the urinary bladder at an angle [2]. Each suprarenal gland is a flat, retroperitoneal endocrine gland sitting on top of the corresponding kidneys. Each suprarenal gland measures approximately 50 mm in height, 30 mm in breadth, and 10 mm in thickness and weighs about 5 grams [3].

Unilateral duplication of the ureter is a common congenital renal abnormality with a prevalence of 0.7 to 4% [4]. The incidence at autopsies is 0.8% [5]. Females are more likely to have renal tract duplication [4]. In most cases, patients are asymptomatic. However, they may present clinically with various symptoms such as vesicoureteral reflux, urinary stones, recurrent urinary tract infection, obstructive uropathy, and ureterocele [6].

Patients with enlarged suprarenal glands may present as back pain or with symptoms of endocrine disorders like Cushing syndrome. They may also present as a mass in the adrenal gland during investigations.

## Case presentation

During the routine dissection classes for the first-year undergraduates, uncommon variation in the left ureter with the enlarged left suprarenal gland was observed in the male cadaver, aged 59 years.

Examination of the anterior and posterior abdominal walls found no pathological or traumatic lesions, including surgical procedure marks. The abdominal cavity was opened through an anterior approach following guidelines of Cunningham's Manual of Practical Anatomy Volume 2: Thorax and Abdomen [7]. The mesentery was identified and dissected, and the small and large intestines were removed to expose the retroperitoneal organs and the related structures. To identify the lower pole of the kidney, the connective tissue was removed.

**Figure 1:**
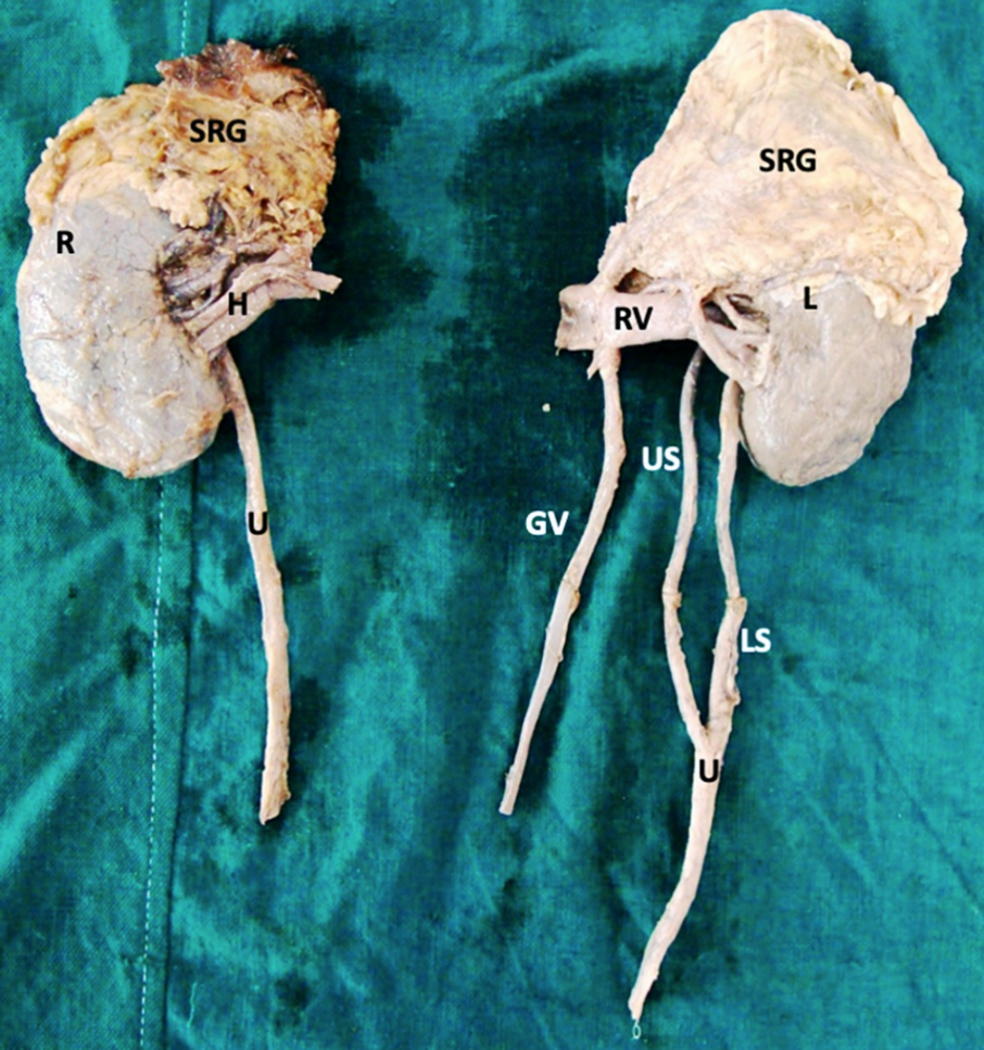
Diagram depicting the duplication of the upper part of left ureter with left enlarged suprarenal gland R- Right; L- Left; SRG- Suprarenal gland; H- Hilum; RV- Renal Vein; U- Ureter; US- upper segment; LS- lower segment; GV- gonadal vein

**Figure 2:**
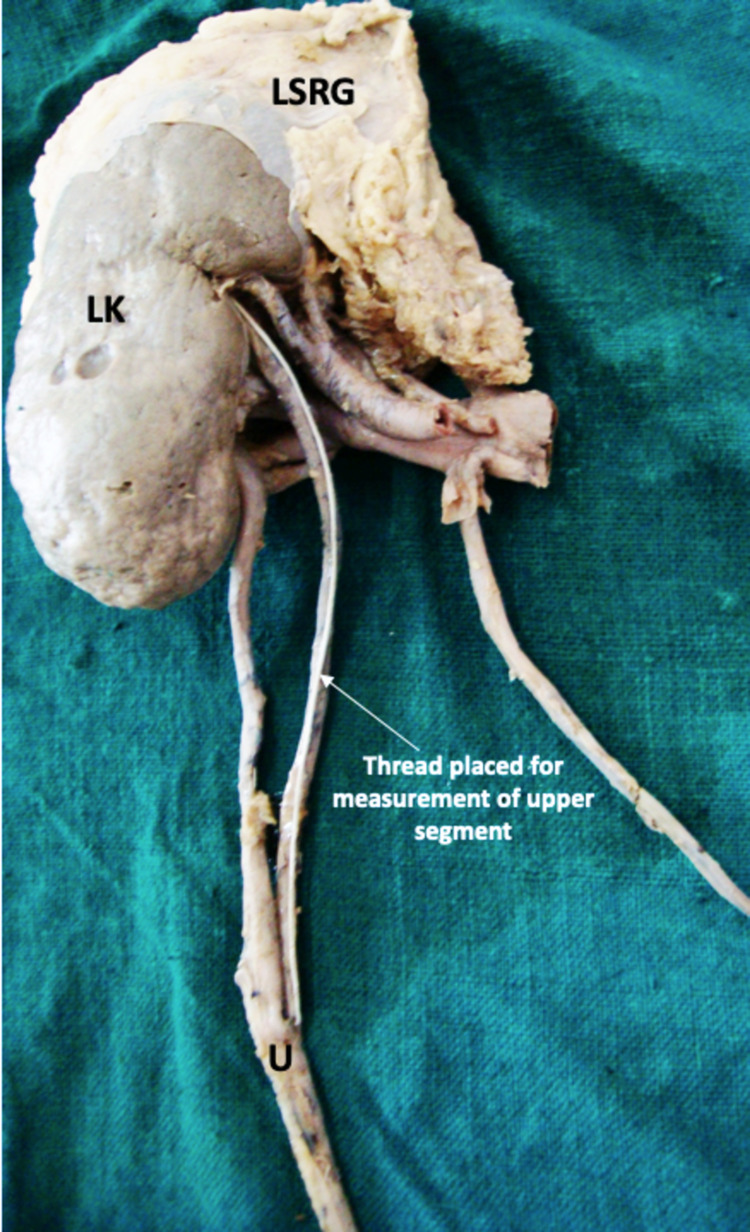
Identification of duplicated left ureter from posterior aspect LK: left kidney, LSRG: left suprarenal gland, U: ureter

The upper part of the left ureter was duplicated. This upper duplicated segment is attached to the kidney at the level of the renal pelvis, while the lower segment is attached below the renal hilum. The average measurements of each segment were 12.5 cm (upper segment) and 10.6 cm (lower segment) (Figure 1, 2).

The left suprarenal gland was enlarged, occupying most of the upper half of the left kidney. The kidney and suprarenal gland's vasculature followed normal branching patterns. The average measurements of the left suprarenal gland are in table 1. No gland or vascular variation was noted on the right side of the same cadaver.

**Table 1:** Measurements of Suprarenal Gland

Measurements	Normal	Left side	Right side
Height	50 mm	70 mm	50 mm
Breadth	30 mm	72 mm	32 mm
Thickness	10 mm	20 mm	10 mm
Weight	5 gm	18 gm	7 gm

## Discussion

The ureter develops during the fifth week of intrauterine life. Pronephros is the most primitive kidney created, followed by the mesonephros with its duct. Finally, metanephros develops. The ureteric bud develops from the mesonephric duct as a diverticulum and invades the nephrogenic blastema. The renal collecting system is formed when this ureteric bud separates into branches. Early division of the ureteric bud extending beyond the renal pelvis results in unilateral ureter duplication [8]. The interaction of the ureteric bud and the metanephric blastema is crucial for the ureter and collecting system of the future kidney to develop properly. PAX2 is expressed in the developing kidney's ureteric bud and differentiating nephrogenic mesenchyme. A bifid ureter is caused by a mutation in this gene [9]. As described in the literature, upper urinary tract abnormalities may be related to gonadal dysgenesis [10]. Additionally, two key factors involved in normal urinary bud growth and elongation are bone morphogenic factor 4 (BMP- 4) and a nephrogenic factor generated from glial cells. Normal renal tract organogenesis requires bone morphogenic factor 4 [11].

In literature, a subject with Acquired Immunodeficiency Syndrome (AIDS) with disseminated histoplasmosis was found to have adrenal masses in the Suprarenal gland. At the same time, it was a malignant peripheral nerve sheath tumor [12]. A female aged 37, complaining of back pain, was investigated to have large bilateral suprarenal masses confirmed as T-cell lymphoma [13]. Lack EE, in his book, explains autopsy findings of 'Adrenal- renal heterotopia' as the entire adrenal gland is located beneath the renal capsule over the superior pole or anterior surface of the kidney [14]. In a neonatal screening program, the incidence of classical congenital adrenal hyperplasia (autosomal recessive inheritance) was 1 in 15000-16000 live births worldwide with significant geographical and ethnic variations. Late-onset congenital adrenal hyperplasia has a higher incidence of 1:1000 worldwide. The reason is an enzymatic deficiency (more than 90% cases are due to 21-hydroxylase enzyme deficiency) in the biosynthesis of cortisol and aldosterone, causing negative feedback, leading to continued adrenocorticotropic hormone (ACTH) secretion. This causes adrenal hyperplasia and androgen excess [15]. Suprarenal gland hyperplasia or neoplasia occurs due to endocrine disorders such as Cushing syndrome, multiple endocrine neoplasia-1. The medulla of suprarenal glands is a frequent site of pheochromocytoma that originates from the medullary cells. Other suprarenal gland enlargement cases are non-functional factors such as inflammation, obesity, or depression [16-18].

Thus, early diagnosis of Ureter duplication abnormality is critical for improved care and survival rates. Understanding the ureter and suprarenal gland variation is essential for surgeons, radiologists, and anatomists.

## Conclusions

As surgeons approach renal surgeries differently, the findings of renal anomalies increase. Damage to the accessory ureter can result in unwelcome complications following surgery. Knowledge of the presence of an accessory ureter and an enlarged suprarenal gland is clinically important for renal surgeons, radiologists, and anatomists.
